# The impact of *CASR* A990G polymorphism in response to cinacalcet treatment in hemodialysis patients with secondary hyperparathyroidism

**DOI:** 10.1038/s41598-021-97587-8

**Published:** 2021-09-09

**Authors:** Jaruwan Ngamkam, Somratai Vadcharavivad, Nutthada Areepium, Titinun Auamnoy, Kullaya Takkavatakarn, Pisut Katavetin, Khajohn Tiranathanagul, Kearkiat Praditpornsilpa, Somchai Eiam-Ong, Paweena Susantitaphong

**Affiliations:** 1 Department of Pharmacy Practice, Faculty of Pharmaceutical Sciences, Chulalongkorn University, Bangkok, 10330 Thailand; 2 Faculty of Pharmaceutical Sciences, Burapha University, Chon Buri, 20131 Thailand; 3 Division of Nephrology, Department of Medicine, Faculty of Medicine, Chulalongkorn University, Bangkok, 10330 Thailand; 4 Excellent Center of Geriatrics, King Chulalongkorn Memorial Hospital, Bangkok, 10330 Thailand

**Keywords:** Chronic kidney disease, Phosphorus metabolism disorders, Genetics research

## Abstract

The objective of this study was to determine the impact of calcium sensing receptor (*CASR*) A990G genetic polymorphism on parathyroid hormone (PTH) lowering response to cinacalcet treatment when controlling for significant influencing clinical factors. This retrospective study was conducted on 135 Thai hemodialysis (HD) patients with secondary hyperparathyroidism (SHPT). *CASR* A990G genotypes were determined. The patients were identified as either G carriers (heterozygous or homozygous *CASR* 990G allele carriers) or noncarriers (homozygous *CASR* 990A carriers). Tested covariates were baseline PTH level (bPTH), baseline serum phosphate (bPhos), baseline serum calcium (bCa), baseline calcitriol equivalent dose (bCtriol), baseline ergocalciferol dose (bErgo), and age. The ANCOVA showed that intact PTH levels after 12 weeks of cinacalcet treatment (PTHw12) was significantly lower among G carriers compared with noncarriers after controlling for bPTH, bPhos, bCtriol, and bErgo (F(1, 127) = 15.472, p < 0.001), with the adjusted mean difference of 253.7 pg/mL. The logistic regression analysis revealed that the odds of a G carrier achieving 30% PTH reduction after 12-week cinacalcet treatment were 3.968 times greater than the odds for a noncarrier after adjusting for bPhos, bCtriol, and age. In conclusion, the *CASR* A990G polymorphism significantly influences cinacalcet response in HD patients with SHPT.

## Introduction

Cinacalcet is an oral calcimimetic agent available for treatment of secondary hyperparathyroidism (SHPT) in chronic kidney disease (CKD). Cinacalcet increases the sensitivity of the calcium-sensing receptor (CASR), the main regulator of PTH secretion, to extracellular ionized calcium ions, resulting in decreased parathyroid hormone (PTH) level^1^. In dialysis patients with SHPT, cinacalcet is efficacious in lowering levels of PTH, serum phosphate, and serum calcium as well as reducing the risks of parathyroidectomy, fractures, and cardiovascular hospitalization^2–11^.

SHPT in CKD is mediated by impaired phosphate excretion, hyperphosphatemia, decreased 1, 25 (OH)_2_ vitamin D, and hypocalcemia. SHPT is present in the majority of hemodialysis (HD) patients and contributes to bone and cardiovascular disorders, which are referred to as chronic kidney disease-mineral bone disorder (CKD-MBD), a serious complication of CKD^12,13^. In dialysis patients, SHPT has been independently associated with all-cause and cardiovascular mortalities^14–17^. Optimal levels of intact parathyroid hormone (iPTH) have been correlated with improved survival, decreased cardiovascular hospitalization, and reduced clinical bone fracture^18–20^. Maintaining iPTH levels in the range of approximately two to nine times the upper limit for the assay used is suggested for dialysis patients^13^.

Cinacalcet significantly reduces plasma levels of PTH, irrespective of the severity of SHPT in dialysis patients^21,22^. A significantly higher percentage of patients achieved plasma PTH target levels. Also, a significantly greater proportion of patients with at least a 30% reduction of plasma PTH levels from baseline levels have been reported among patients who were treated with cinacalcet, compared with those who received control treatment^4,10,11,22–24^. However, interpatient variability in response has been observed and not all patients have a good response to cinacalcet therapy. A reduction of PTH levels after three months of treatment with relatively lower doses has been reported as a prognostic marker of good response to cinacalcet treatment^25^.

Several factors, including high baseline PTH, persistent hyperphosphatemia, and nodular hyperplasia of parathyroid glands have been reported to significantly influence the response to cinacalcet^25,26^. Additionally, the association between the A990G SNP of the gene encoding calcium-sensing receptor (CASR) and the biochemical response to cinacalcet treatment has been observed in two previous studies^27,28^. Rothe et al.^27^ reported the findings in seven HD patients with SHPT and first proposed the possibility that the *CASR* A990G genetic polymorphism may be one of the possible causes of significant interpatient differences in the response to cinacalcet treatment. Later, Jeong et al.^28^ observed the association between *CASR* A990G polymorphism and reduction of iPTH during a 3-month cinacalcet treatment among 70 HD patients with SHPT. On the contrary, Moe et al.^29^ found no association between the *CASR* A990G SNP and change of iPTH from baseline to 20 weeks after cinacalcet treatment in HD patients of European and African ancestry.

Available information regarding the influence of *CASR* A990G genetic polymorphism on cinacalcet response is inconclusive and needs to be explored. To determine the impact of the *CASR* A990G polymorphism on PTH lowering effect of cinacalcet in HD patients with SHPT, the primary objective of this study was to compare iPTH levels after 12 weeks of the treatment (PTHw12) between *CASR* 990G carrier and noncarrier genotypes while considering the variability of other clinical factors that significantly influence PTH level. The secondary objective was to assess the effect of the polymorphism on achievement of at least 30% reduction of iPTH levels from their baselines, after 12-week cinacalcet treatment, comparing between the two genotype groups while controlling for significant factors.

## Results

### Characteristics of the study population

**Figure 1:**
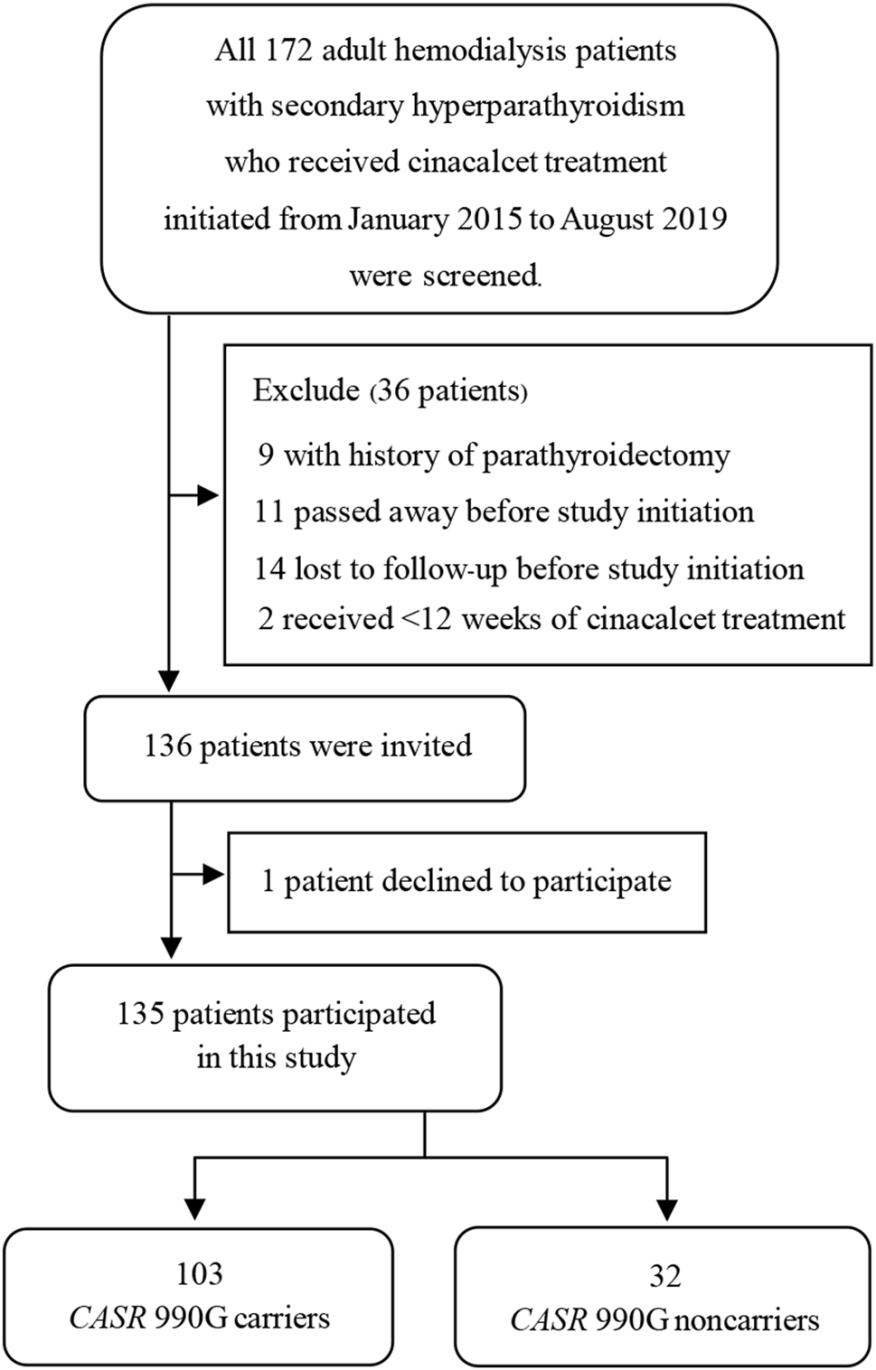
Flow diagram of the inclusion and exclusion process.

A total of 135 Thai HD patients with SHPT participated in this study (Fig. 1). The characteristics of the participants are summarized in Table 1. Almost all participants (98.5%) had severe SHPT (iPTH > 800 pg/mL). At the time of starting cinacalcet, 18 patients (13.3%) had been receiving one phosphate binding agent and 96 patients (71.1%) had been receiving two phosphate binders. There were 35 (25.9%) and 37 (27.4%) patients who had been prescribed calcitriol and alfacalcidol, respectively. Seventy-two patients (53.3%) had been prescribed ergocalciferol.

**Table 1:** Baseline characteristics and medication use on the day of starting cinacalcet treatment.

Characteristics^a^	Total (n = 135)	G carriers (n = 103)	G noncarriers (n = 32)	p value^b^
Male, n (%)	66 (48.9)	48 (46.6)	18 (56.3)	0.340
Body weight, kg	56.0 (49.8, 66.5)	54.9 (49.8, 64.4)	61.3 ± 15.1	0.380
Age, years	59.5 ± 13.2	58.8 ± 13.3	61.7 ± 12.8	0.273
Dialysis duration, years	4.0 (2.4, 6.0)	4.0 (2.4, 6.0)	4.3 ± 2.9	0.752
Dialysate calcium, mEq/L	2.5 (2.5, 3.0)	2.5 (2.5, 3.0)	2.5 (2.5, 3.0)	0.200
Serum iPTH, pg/mL	1262.0 (1018.0, 1633.0)	1237.0 (990.1, 1622.0)	1390.0 ± 336.6	0.152
Serum calcium^c^, mg/dL	9.7 (9.3, 10.3)	9.8 ± 0.8	9.9 ± 0.8	0.483
Serum phosphate, mg/dL	4.7 ± 1.4	4.7 ± 1.5	4.7 ± 1.2	0.934
Serum ALP, U/L	144.0 (103.0, 240.0)	139.0 (102.0, 226.0)	185.0 (110.0, 255.0)	0.159
Serum creatinine, mg/dL	9.2 (7.8, 11.2)	9.2 (7.8, 11.0)	9.2 (7.9, 11.5)	0.610
Serum 25-(OH)D^d^, ng/mL	25.5 (19.4, 33.4)	25.5 (19.6, 33.8)	25.3 ± 7.8	0.313
**Medications:**				
**Phosphate binders**				
Calcium carbonate, n (%)	36 (26.7)	31 (30.1)	5 (15.6)	0.106
Lanthanum carbonate, n (%)	60 (44.4)	42 (40.8)	18 (56.3)	0.124
Sevelamer carbonate, n (%)	32 (23.7)	24 (23.3)	8 (25.0)	0.844
Aluminium hydroxide, n (%)	4 (3.0)	4 (3.9)	0	0.258
**Calcitriol & vitamin D analog**				
Calcitriol, n (%)	35 (25.9)	26 (25.2)	9 (28.1)	0.745
Calcitriol dose, μg/week	1.5 (0.8, 3.0)	1.5 (0.8, 3.0)	2.3 (1.0, 4.0)	0.392
Alfacalcidol, n (%)	37 (27.4)	30 (29.1)	7 (21.9)	0.422
Alfacalcidol dose, μg/week	2.3 (1.5, 3.8)	3.0 (1.5, 4.0)	1.5 (1.5, 2.3)	0.123
**Vitamin D**				
Ergocalciferol, n (%)	72 (53.3)	54 (52.4)	18 (56.3)	0.705
Ergocalciferol dose, *20,000 IU/month	4.0 (4.0, 12.0)	4.0 (4.0, 10.0)	4.0 (4.0, 12.0)	0.595

*iPTH* intact parathyroid hormone, *ALP* alkaline phosphatase, *25-(OH)D* 25-hydroxy vitamin D.

^a^Continuous data are expressed with either means ± standard deviations if they are normally distributed or medians (interquartile ranges) if they are not normally distributed.

^b^The p values were calculated with the use of the chi-square test, the Mann–Whitney U test, or the t-test for comparison between G carriers and noncarriers.

^c^Serum calcium is reported as albumin corrected values if serum albumin was less than 4.0 g/dL [corrected calcium in mg/dL = measured calcium in mg/dL + 0.8*(4—serum albumin in g/dL)].

^d^Serum 25-(OH)D was that of 126 patients only.

Of 135 participants, there was a significant median (interquartile ranges, IQR) decrease in iPTH level 1262.0 (1018.0, 1633.0) pg/mL from baseline to 602.0 (437.1, 1053.0) pg/mL at week 12 of cinacalcet treatment (Wilcoxon Signed Rank test, p < 0.001); a median (IQR) reduction of 521.6 (286.0, 993.4) pg/mL, with the corresponding median (IQR) percentage of iPTH reduction from baseline of 49.8% (21.5%, 67.3%). A reduction in plasma iPTH of at least 30% from baseline was observed in 79 patients (58.5%). Sixty-four patients (47.4%) achieved the KDIGO recommended target of two to nine times the upper limit for the PTH assay used for CKD patients on dialysis (130–585 pg/mL). The iPTH of 15 patients (11.1%) increased from baseline even after 12-week cinacalcet treatment.

The allele frequencies of *CASR* 990A and 990G were 47.8% and 52.2%, respectively. The genotype frequencies were in accordance with the Hardy–Weinberg equilibrium (chi-square = 0.186, p = 0.911). The AA, AG, and GG genotypes were observed in 32 (23.7%), 65 (48.1%), and 38 (28.1%) patients, respectively. Therefore, 103 patients (76.3%) were identified as G carriers and 32 (23.7%) were noncarriers. Baseline characteristics were comparable between the two groups.

**Figure 2:**
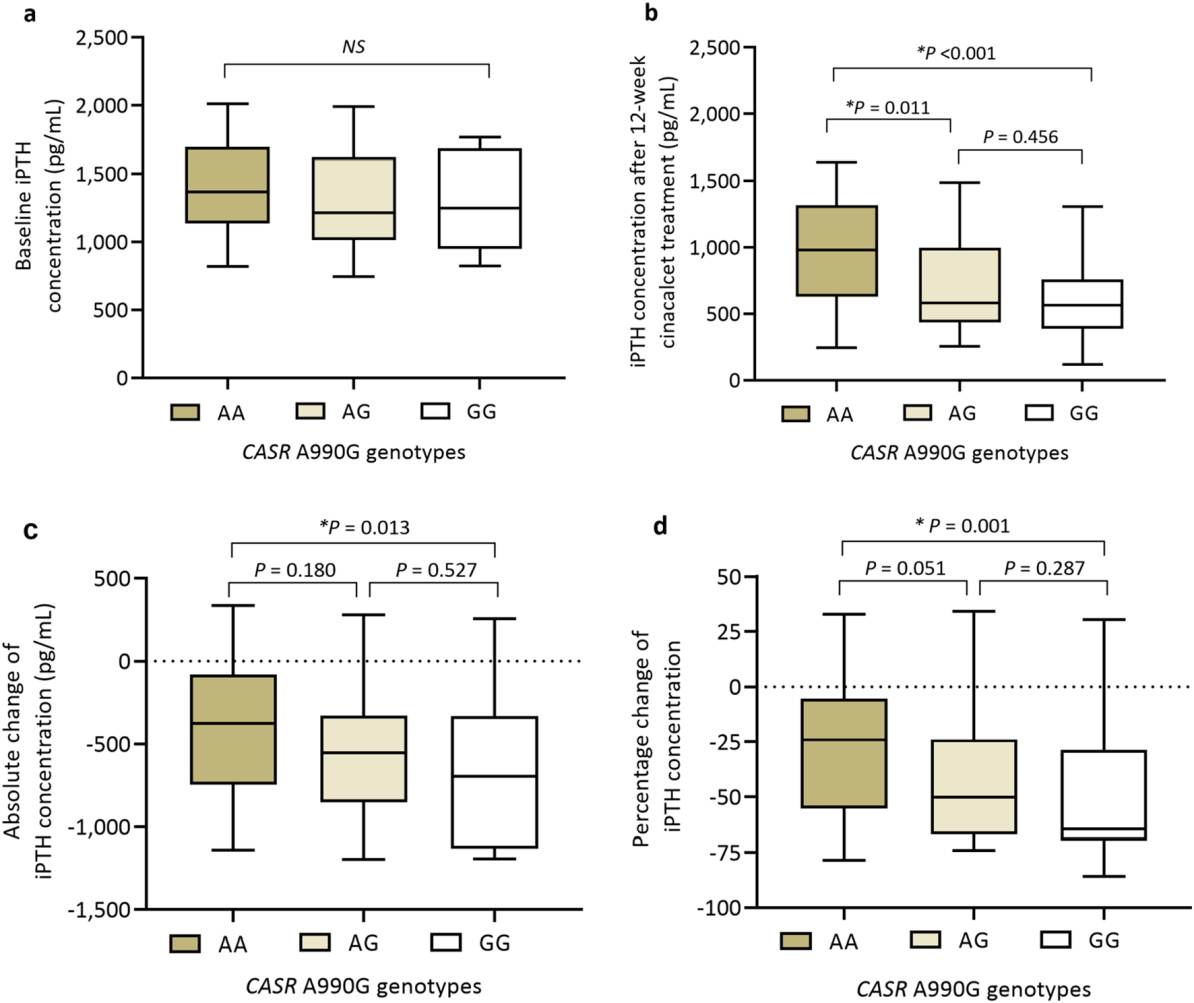
Box plots of intact parathyroid hormone (iPTH) concentrations before and after 12 weeks of cinacalcet treatment of 135 participants (**a**) Baseline iPTH data (**b**) iPTH concentration after 12 weeks of cinacalcet treatment (**c**) Absolute change of iPTH concentration from baseline to 12 weeks (**d**) Percentage change of iPTH concentration from baseline to 12 weeks. In each box, the top, the middle line, and the bottom represent the 75th percentile, the median, and the 25th percentile, respectively. The whiskers show the maximum and the minimum. The data were compared by Kruskal–Wallis test among three genotypes and by Mann–Whitney test between two genotypes. *p < 0.05 was considered statistically significant.

Absolute change and percentage change of iPTH levels from baseline to after 12 weeks of cinacalcet treatment among the three genotype groups are presented in Fig. 2. Of 15 patients in whom PTHw12 increased from baseline, the AA, AG, and GG genotypes were observed in seven (46.7%), five (33.3%), and three (20.0%) patients, respectively. The median (IQR) reduction of iPTH levels and the corresponding median (IQR) percentage reduction of iPTH levels from baseline were significantly higher in G carriers than those in noncarriers as shown in Table 2.

**Table 2:** Serum parathyroid hormone, serum calcium, serum phosphate and medication use after 12 weeks of cinacalcet treatment.

Parameters^a^	Totals **(**n = 135)	G carriers (n = 103)	G noncarriers (n = 32)	p value^b^
**Serum iPTH levels:**				
PTHw12, pg/mL	602.0 (437.1, 1053.0)	577.2 (417.9, 907.2)	979.4 ± 390.0	< 0.001
PTH reduction, pg/mL	521.6 (286.0, 993.4)	578.0 (330.0, 1024.7)	416.9 ± 438.4	0.012
Percentage of PTH reduction, %	49.8 (21.5, 67.3)	57.8 (25.6, 68.1)	27.5 ± 30.2	0.002
Proportion of patients achieved at least 30% PTH reduction, n (%)	79 (58.5)	66 (64.1)	13 (40.6)	0.019
Proportion of patients achieved the KDIGO target^c^, n (%)	64 (47.4)	57 (55.3)	7 (21.9)	0.001
Serum calcium^d^, mg/dL	9.1 ± 0.9	9.1 ± 0.9	9.3 ± 0.8	0.200
Serum phosphate, mg/dL	4.0 (3.3, 4.9)	3.9 (3.2, 4.9)	4.3 (3.4, 4.9)	0.309
**Medications use:**				
**Phosphate binders**				
Calcium carbonate, n (%)	39 (28.9)	34 (33.0)	5 (15.6)	0.058
Lanthanum carbonate, n (%)	60 (44.4)	42 (40.8)	18 (56.3)	0.124
Sevelamer carbonate, n (%)	32 (23.7)	24 (23.3)	8 (25.0)	0.844
Aluminium hydroxide, n (%)	4 (3.0)	4 (3.9)	0	0.258
**Calcitriol & vitamin D analog**				
Calcitriol, n (%)	36 (26.7)	26 (25.2)	10 (31.3)	0.500
Calcitriol dose, μg/week	1.5 (1.0, 3.0)	1.5 (1.25, 3.0)	2.3 (1.0, 4.0)	0.419
Alfacalcidol, n (%)	39 (28.9)	31 (30.1)	8 (25.0)	0.578
Alfacalcidol dose, μg/week	2.3 (1.5, 3.8)	3.0 (1.5, 4.0)	1.5 (1.5, 2.3)	0.123
Vitamin D				
Ergocalciferol, n (%)	70 (51.9)	52 (50.5)	18 (56.3)	0.569
Ergocalciferol dose, *20,000 IU/month	4.0 (4.0, 12.0)	4.0 (4.0, 11.0)	4.0 (4.0, 12.0)	0.480

*iPTH* intact parathyroid hormone, *PTHw12* intact parathyroid hormone levels after 12 weeks of cinacalcet treatment.

^a^Continuous data are expressed with either means ± standard deviations if they are normally distributed or medians (interquartile ranges) if they are not normally distributed.

^b^The p values were calculated with the use of the Mann–Whitney U test, or the t-test for comparison between G carriers and noncarriers.

^c^The KDIGO recommended target of two to nine times the upper limit for the PTH assay used for CKD patients on dialysis (130–585 pg/mL).

^d^Serum calcium is reported as albumin corrected values if serum albumin was less than 4.0 g/dL [corrected calcium in mg/dL = measured calcium in mg/dL + 0.8*(4—serum albumin in g/dL)].

### Association between *CASR* genotypes and PTHw12

Analysis of covariance (ANCOVA) was used to compare PTHw12 between G carriers and noncarriers. Initial data screening led to the logarithmic transformation of PTHw12 for conforming data to the normal distribution (Kolmogorov–Smirnov test, p = 0.098). No strong correlation was found among potential covariates (*r* ranged from − 0.262 to 0.175). There were significant linear relationships between log PTHw12 and baseline PTH level (bPTH), baseline serum calcium concentration (bCa), baseline calcitriol dose or relative calcitriol equivalent dose of alfacalcidol (bCtriol), and baseline ergocalciferol dose (bErgo) (all p < 0.05). The trends of linear relationships between log PTHw12 and the other two factors, baseline serum phosphate concentration (bPhos) and age of patients (AGE), were also observed (p = 0.059 and 0.055, respectively); therefore, all six factors were tested as a covariate.

With all test assumptions fulfilled, the ANCOVA revealed that there was a significant difference in log PTHw12 between G carriers and noncarriers after controlling for significant covariates (F(1, 127) = 15.472, p < 0.001, ŋ_p_^2^ = 0.109). The significant covariates were bPTH, bCtriol, bPhos, and bErgo while AGE and bCa were not significant. The adjusted mean difference of the untransformed PTHw12 between G carriers and noncarriers was 253.7 pg/mL after controlling for significant covariates. The observed and adjusted mean values of log PTHw12, together with untransformed PTHw12 among *CASR* genotypes, are presented in Table 3.

**Table 3:** ANCOVA results and descriptive statistics for log PTHw12 (n = 135).

Log PTHw12 (pg/mL)	Observed mean	Adjusted mean^a^	SD	95% CI^b^		Untransformed PTHw12^c^
				Lower	Upper	
*CASR* G carrier (n = 103)	2.78	2.78	0.23	2.74	2.83	605.3
*CASR* G noncarrier (n = 32)	2.95	2.93	0.21	2.87	2.99	859.0

Source	Sum of squares	df	Mean square	F	p value	Partial eta-squared
*CASR* polymorphism	0.550	1	0.550	15.472	< 0.001	0.109
bPTH (*100 pg/mL)	0.785	1	0.785	22.095	< 0.001	0.148
bPhos (mg/dL)	0.267	1	0.267	7.515	0.007	0.056
bCa (mg/dL)	0.089	1	0.089	2.506	0.116	0.019
bCtriol (μg/week)	0.449	1	0.449	12.646	0.001	0.091
bErgo (*20,000 IU/month)	0.174	1	0.174	4.902	0.029	0.037
AGE (years)	0.131	1	0.131	3.673	0.058	0.028

R^2^ = 0.382 (adjusted R^2^ = 0.348); The homogeneity of regression slopes was evaluated by conducting a preliminary univariate ANOVA, which indicated that there were no significant interactions between each of the tested covariates and the *CASR* polymorphisms (all p > 0.05). Levene’s test reveals equal variances for each group of measurements, F(1, 133) = 1.442, p = 0.232. Log PTHw12 in years = 2.358—0.152 (if G carrier) + 0.023 (bPTH in *100 pg/mL) + 0.33 (bPhos in mg/dL) + 0.034 (bCa in mg/dL)—0.042 (bCtriol in μg/week)—0.009 (bErgo in *20,000 IU/month)—0.002 (AGE in years).

*AGE* age on the day of starting cinacalcet, *bCa* baseline serum calcium concentration, *bCtriol* baseline calcitriol dose or relative calcitriol equivalent dose of alfacalcidol, *bErgo* ergocalciferol dose before starting cinacalcet, *bPhos* baseline serum phosphate concentration, *bPTH* baseline serum intact parathyroid hormone concentration, *PTHw12* intact parathyroid hormone levels after 12 weeks of cinacalcet treatment.

^a^Adjusted mean values based on bPTH = 13.1958 *100 pg/mL, bPhos = 4.6815 mg/dL, bCa = 9.8080 mg/dL, bCtriol (0 = no use) = 1.0426 μg/week, bErgo (0 = no use) = 3.4978 *20,000 IU/month, and AGE = 59.4728 years.

^b^95% confidence interval from bootstrapping of adjusted mean values, based on 5000 bootstrap samples.

^c^The untransformed PTHw12 calculated by back-transforming the adjusted mean values.

Significant differences in log PTHw12 were also found among the three genotype groups after controlling for bPTH, bCtriol, bPhos, and bErgo [F(2,126) = 8.817 (p < 0.001, ŋ_p_^2^ = 0.123]. Multiple comparisons revealed a significantly higher adjusted mean of log PTHw12 in AA group compared with those in AG and GG groups (p = 0.005 and < 0.001, respectively); however, adjusted log PTHw12 means were comparable between AG and GG groups (p = 0.468). The AA group had the highest untransformed PTHw12 (859.0 pg/mL) compared with AG and GG groups (635.3 and 555.9 pg/mL, respectively).

### Association between *CASR* genotypes and achievement of 30% PTH reduction

**Figure 3:**
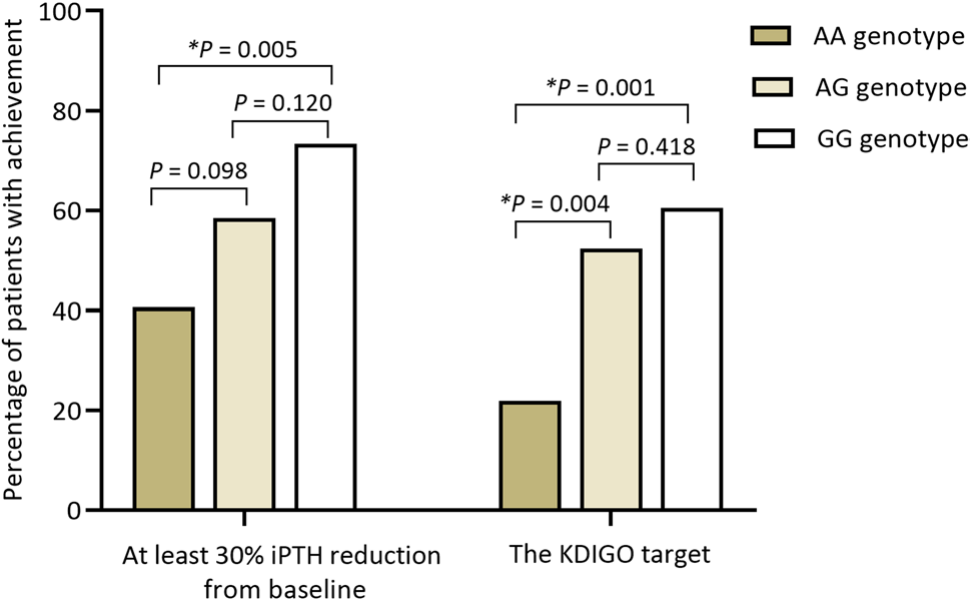
The percentage of patients that achieved at least 30% reduction of intact parathyroid hormone concentration from baseline and the KDIGO recommended PTH target of two to nine times upper limit for the assay used for dialysis patients (130–585 pg/mL) after 12 weeks of cinacalcet treatment. The data were compared by chi-square test. *p < 0.05 was considered statistically significant.

The proportions of patients who achieved at least 30% reduction of iPTH in three genotype groups are shown in Fig. 3. The characteristics of patients who achieved or did not achieve iPTH reduction of at least 30% are summarized in Table 4.

**Table 4:** Characteristics of patients in groups classified by the achievement of at least 30% parathyroid hormone reduction form baseline after 12 weeks of cinacalcet treatment (n = 135).

Parameters^a^	Parathyroid hormone reduction from baseline		p value^b^
	At least 30% reduction (n = 79)	Less than 30% reduction (n = 56)	
*CASR* genotypes			0.019
G carriers, n (%)	66 (64.1)	37 (35.9)	
G noncarriers, n (%)	13 (40.6)	19 (59.4)	
bPTH (*100 pg/mL)	1277.0 (1025.0, 1629.0)	1189.5 (983.3, 1688.6)	0.646
bPhos (mg/dL)	4.4 ± 1.3	5.0 ± 1.5	0.013
bCa (mg/dL)	9.6 (9.2, 10.1)	10.0 ± 0.8	0.016
bCtriol (μg/week)	2.0 (1.5, 3.0)	1.0 (0.8, 1.8)	0.001
bErgo (*20,000 IU/month)	8.0 (4.0, 12.0)	4.0 (4.0, 8.0)	0.206
AGE (years)	62.4 ± 12.8	55.3 ± 12.7	0.002

*AGE* age on the day of starting cinacalcet, *bCa* baseline serum calcium concentration, *bCtriol* baseline calcitriol dose or relative calcitriol equivalent dose of alfacalcidol, *bErgo* ergocalciferol dose before starting cinacalcet, *bPhos* baseline serum phosphate concentration, *bPTH* baseline serum intact parathyroid hormone concentration.

^a^Continuous data are expressed with either means ± standard deviations if they are normally distributed or medians (interquartile ranges) if they are not normally distributed.

^b^The p values were calculated with the use of the chi-square test, the Mann–Whitney U test, or the t-test for comparison between G carriers and noncarriers.

A binomial logistic regression was performed to assess the impact of *CASR* polymorphism on the likelihood of patients having at least 30% reduction of iPTH from their baseline levels after 12 weeks of cinacalcet treatment when controlling for significant influencing factors. The logistic regression coefficient, Wald test, and odds ratio for each of the tested variables are presented in Table 5.

**Table 5:** Logistic regression analysis results (n = 135).

Predictor	β	SE β	Wald	df	p value	Odds ratio	95% CI^a^	
							**lower**	**upper**
Constant	0.868	3.578	0.059	1	0.808	2.381	–	–
*CASR* genotype (1 = G carrier, 0 = noncarrier)	1.378	0.529	6.786	1	0.009	3.968	1.262	21.179
bPTH (*100 pg/mL)	0.039	0.065	0.352	1	0.553	1.039	0.896	1.241
bPhos (mg/dL)	-0.388	0.168	5.309	1	0.021	0.679	0.483	0.869
bCa (mg/dL)	-0.426	0.288	2.199	1	0.138	0.653	0.319	1.134
bCtriol (μg/week)	0.772	0.226	11.668	1	0.001	2.165	1.357	4.899
bErgo (*20,000 IU/month)	0.065	0.053	1.511	1	0.219	1.067	0.945	1.249
AGE (years)	0.052	0.018	8.284	1	0.004	1.053	1.014	1.121

The dependent variable in this analysis is achievement of at least 30% reduction of PTH from baseline value (1 = at least 30% PTH reduction and 0 = less than 30% PTH reduction from baseline); Predicted logit of achieving 30% PTH reduction = 0.868 + 1.378 (if G carrier) + 0.039 (bPTH in *100 pg/mL)—0.388 (bPhos in mg/dL)—0.426 (bCa in mg/dL) + 0.772 (bCtriol in μg/week) + 0.065 (bErgo in *20,000 IU/month) + 0.052 (AGE in years); All tested continuous variables were linearly related to the logit of the dependent variable (Box-Tidwell test, all p > 0.05); Omnibus test of model coefficients, chi-square = 45.940, df = 7, p < 0.001; Nagelkerke R square = 0.388; Hosmer and Lemeshow test, chi-square = 14.753, df = 8, p = 0.064; With the cutoff set at 0.5 for prediction of 30% PTH reduction achievement, the model was able to correctly classify 79.7% of those who achieved at least 30% iPTH reduction and 62.5% of those who did not, for the overall correction prediction of 72.6%

*β* the partial logistic regression coefficient, *SE β* the standard errors of the partial slope coefficient, *df* degree of freedom, *AGE* age on the day of starting cinacalcet, *bCa* baseline serum calcium concentration, *bCtriol* baseline calcitriol dose or relative calcitriol equivalent dose of alfacalcidol, *bErgo* ergocalciferol dose before starting cinacalcet, *bPhos* baseline serum phosphate concentration, *bPTH* baseline serum intact parathyroid hormone concentration.

^a^95% confidence interval of the odds ratio from bootstrapping, based on 5000 bootstrap samples.

With all test assumptions fulfilled, the regression model indicated that partial effects of the genotype, bPhos, bCtriol and AGE were significant, while influences of bPTH, bCa, and bErgo were not. The regression coefficient for genotype was 1.378; the genotype was coded as either 1 = G carriers or 0 = noncarriers. When holding all other variables constant, the odds of a G carrier achieving 30% iPTH reduction after 12 weeks of cinacalcet treatment were 3.968 [bootstrap 95% confidential interval (CI): 1.262 to 21.179] times greater than the odds for a noncarrier. In addition, the log of the odds of achieving 30% iPTH reduction was positively related to bCtriol and AGE, but negatively associated with bPhos.

## Discussion

In this study, the impact of *CASR* polymorphism on the efficacy of 12-week cinacalcet treatment, in terms of PTH reduction, was determined in adult HD patients with SHPT in real clinical practice. There were 135 participants, of which 103 were G carriers and 32 were noncarriers, representing 76% and 24% of the study population, respectively. Despite similar baseline characteristics, G carriers had significantly lower PTH levels compared with noncarriers after 12 weeks of cinacalcet treatment, with the adjusted mean difference of the untransformed PTH levels of 253.7 pg/mL, when controlling for significant influencing factors, namely, bPTH, bPhos, bCtriol, and bErgo. Furthermore, the odds of a G carrier achieving at least a 30% reduction in PTH concentrations from baseline to week 12 were approximately four times higher than those for a noncarrier, after adjusting for significant covariates, namely, bPhos, bCtriol, and AGE.

The CASR, a G protein-coupled receptor highly expressed in the parathyroid chief cells, plays an important role as the key modulator of parathyroid gland function by sensing perturbations of extracellular ionized calcium concentration from its narrow physiological range, and regulating PTH secretion, synthesis, and degradation, as well as parathyroid cellular proliferation^30^. Activation of the CASR by increased extracellular Ca^2+^ stimulates multiple G proteins and confers an intracellular signaling cascade, which leads to inhibition of PTH secretion. Cinacalcet, a type II calcimimetic agent, acts as a positive allosteric modulator of the CASR. By interacting with the seven-transmembrane domain of the CASR, cinacalcet lowers the threshold for receptor activation by extracellular Ca^2+^, presumably by inducing conformational change in the receptor, thereby promoting activation of signal transduction pathways leading to reduction of the circulating PTH level at normal, or even at low, serum calcium levels^31^. The *CASR* A990G SNP alters the amino acid sequence and changes the conformation of the CASR intracellular carboxyl-terminal domain, altering its co-operative response to Ca^2+^ and its binding to filamin-A, a scaffold protein for a signaling cascade^32,33^. It has also been demonstrated that the glycine substitution at arginine-990 encoded by the *CASR* A990G SNP results in a gain-of-function of the CASR^34^. Although the precise underlying mechanism needs to be further explored, the greater PTH lowering effect of cinacalcet observed in *CASR* 990G carriers compared with noncarriers could be explained by the combined effect of the *CASR* A990G polymorphism and cinacalcet treatment.

Genetic polymorphisms of several genes related to PTH regulation have been considered as plausible factors influencing cinacalcet response. Three nonsynonymous variants at the intracellular domain of the CASR encoded by *CASR* A986S (rs1801725), *CASR* Q1011E (rs1801726), and *CASR* A990G (rs1042636) have been identified. The frequency of these polymorphisms varies between different populations. *CASR* A986S is the most common SNP of the *CASR* gene among Caucasians, and *CASR* Q1011E is a common polymorphism in populations with African ancestry, whereas *CASR* A990G occurs at high frequency in Asians^35^. It has been reported that linkage disequilibrium exists among them, indicating that these SNPs are not independent and are highly correlated^36^. Of note, the frequency of occurrence of both *CASR* A986S and *CASR* Q1011E is quite low among Asians, likely limiting the statistical power to detect associations with cinacalcet response (if one exists). In addition, the association between 25 selected SNPs in genes related to calcium regulation, serum phosphate concentration, and bone metabolism were evaluated in Korean SHPT patients on dialysis by Jeong et al^28^. *CASR* A990G and *CASR* rs1802757 were found to be associated with PTH reduction after controlling for age, sex, and baseline calcium level, while no correlations of the tested SNPs, other than *CASR* with PTH reduction during 3-month cinacalcet treatment, were observed. Linkage disequilibrium test showed high correlation between *CASR* A990G and *CASR* rs1802757 (r^2^ = 0.68) and it was mentioned that the functionality of the *CASR* rs1802757, which is located in 3’ untranslated regions of the gene, has not been well documented^28^. Furthermore, the associations of 18 SNPs in *CASR* and one SNP in the vitamin D receptor gene (*VDR)* with response to cinacalcet were examined in another study by Moe et al.^29^. A modest association of the *CASR* rs937627 genotype with percentage change of PTH in the European ancestry population was observed, but no significant interaction with cinacalcet treatment was found. With consideration to the frequency and functionality of polymorphisms together with the correlation of SNPs due to linkage disequilibrium, the polymorphisms other than the *CASR* A990G were, therefore, not assessed in the present study.

The *CASR* 990G allele frequency of 52% in the study patients was similar to that reported among other Asian populations, but higher than those observed among Caucasians and African Americans^35,37–39^. The differences in the 990G allele frequencies observed among ethnicities is a possible explanation for the conflicting results of previous studies assessing the associations between the *CASR* A990G polymorphism and biochemical response of cinacalcet^27–29^.

Although almost all of the patients in this study had very high bPTH of more than 800 pg/mL, a significant reduction of PTH was observed, with the overall median percentage of PTH reduction from baseline to week 12 of approximately 50%. Cinacalcet efficacy in decreasing mean PTH, regardless of severity of SHPT at baseline, is supported by a previous randomized controlled trial. Lindberg et al.^11^ demonstrated that, among 260 HD treated patients (38% white race and 39% black race), the percentage reduction in mean PTH from baseline was 47% in patients with mild SHPT (baseline iPTH, 300–500 pg/mL), 44% in patients with moderate SHPT (baseline iPTH, 501–800 pg/mL), and 33% in patients with severe SHPT (baseline iPTH, more than 800 pg/mL). Comparing those with the same SHPT severity, our studied patients had a higher median percentage of PTH reduction than that reported by Lindberg et al.^11^; this might also be at least partly due to differences in ethnic proportions of *CASR* 990G carriers.

Not only were reductions of PTH from baseline observed, but also the proportions of patients with at least 30% reduction of PTH and the proportions of patients who achieve the KDIGO target range of two to nine times the upper limit for the PTH assay were found to be statistically significant between the two groups of genotypes in this study. Of G carriers, 64% had at least 30% PTH reduction, and 55% achieved the KDIGO recommended PTH target of two to nine times the upper limit for the assay used for dialysis patients, while 40% of noncarriers had at least a 30% PTH reduction, and only 22% achieved the KDIGO recommended PTH target. Moreover, increased iPTH from baseline after 12-week cinacalcet treatment was observed in 7.8% of G carriers and 21.9% of noncarriers. These findings support that *CASR* genetic polymorphism significantly influences the response to cinacalcet treatment.

Notably, although the median baseline PTH level of 1262 pg/mL was observed in this study, the overall proportion of patients who achieved a reduction in PTH of at least 30% from baseline was comparable to results reported in two previous randomized clinical studies, with median baseline PTH levels of approximately 600 to 850 pg/mL^11,24^ but lower than the reported 90.3% among patients randomized to cinacalcet in a phase 3 trial among Japanese HD patients, with median baseline PTH levels of 606.5 pg/mL^10^. This could possibly be explained by differences in baseline PTH levels and ethnicities of the study populations.

Along with the association between *CASR* polymorphism and cinacalcet response, lower PTH, lower phosphate, higher calcitriol or vitamin D analog dosage, and higher ergocalciferol dosage at the time of cinacalcet initiation were found to be significantly associated with lower PTH after a 12-week cinacalcet treatment, while lower phosphate, higher calcitriol or vitamin D analog dosage, and increasing age at the time of cinacalcet initiation were significantly associated with improving the odds of achieving at least 30% PTH reduction. In concordance with our results, the association of multiple clinical factors, including the severity of SHPT, age, race, serum phosphate, and serum calcium, with PTH control among HD patients treated with cinacalcet has been reported^40,41^.

The influence of bPTH on the 30% reduction of PTH was not statistically significant, whereas the significant impact of bPTH on PTHw12 was observed in this study. These findings indicate that, although cinacalcet is efficacious at lowering PTH levels irrespective of the baseline severity of SHPT, the higher the baseline PTH concentrations, the higher are the PTH levels after 12 weeks of cinacalcet treatment. In agreement with our findings, it has also been reported that among Japanese HD patients randomized to cinacalcet^10^, and in a post hoc analysis of pooled data from three randomized controlled trials in dialysis patients^21^, as baseline PTH levels increased, the target PTH of 250 pg/mL was achieved in a lower percentage of patients. Similarly, a recent study in HD patients enrolled in the Dialysis Outcomes and Practice Pattern Study (DOPPS) (Phases 4–6) reported that increased PTH immediately prior to HD initiation predicted a higher PTH level 9–12 months later^40^ and the severity of SHPT was found to be an important clinical factor in determining the response to cinacalcet therapy among HD patients in a real-life observational study^41^.

SHPT is known to be a compensatory response to phosphate retention. The direct effects of phosphate on PTH secretion and parathyroid cell proliferation have long been demonstrated in experiment studies^42^. The significance of bPhos influence on cinacalcet response in terms of mean absolute PTH levels and the proportion of patients achieving at least 30% PTH reduction from baseline was determined in this study. According to the regression model, for each 1 mg/dL increase in bPhos, there was a 32% decrease in the odds of achieving a reduction in PTH of at least 30% after 12 weeks of cinacalcet treatment, when all other factors were held constant.

Augmented effects of combination use of cinacalcet with calcitriol or vitamin D analogs for lowering PTH that were observed in this study have also been reported in previous randomized controlled trials^4,11^. Cinacalcet and active vitamin D sterols have different receptor sites where they exert their pharmacological effects. The possibility of a crossed positive, bidirectional and additive interaction between calcimimetics and vitamin D sterols, probably mediated by the heterologous upregulation of the parathyroid CASR and vitamin D receptors, has been described in experimental conditions^43,44^. In addition, cinacalcet and active vitamin D have disparate effects on calcium and phosphate levels. The use of calcitriol or vitamin D analogs, particularly in a high dose or in combination with calcium-containing phosphate binders, may lead to hypercalcemia and hyperphosphatemia as a result of increased intestinal absorption of calcium and phosphate. On the other hand, cinacalcet suppresses PTH while simultaneously reducing serum calcium and phosphate in dialysis patients. Combination usage of cinacalcet and vitamin D has enabled simultaneous attainment of all four KDOQI bone and mineral targets, which has been reported to improve survival in incident HD patients^45^. Of note, in the present study, the observed serum calcium levels were comparable between G carriers and noncarriers not only at baseline (9.8 ± 0.8 and 9.9 ± 0.8 mg/dL, respectively; p = 0.483) but also after 12 weeks of cinacalcet treatment (9.1 ± 0.9 and 9.3 ± 0.8 mg/dL, respectively; p = 0.200).

The dose of ergocalciferol that the patients had been receiving at the time of initiating cinacalcet treatment also significantly influenced PTHw12 in our studied patients. Ergocalciferol is effective in repleting vitamin D levels in HD patients, though there is no conclusive evidence to support a benefit of vitamin D replacement in dialysis patients. The KDIGO guidelines, however, suggest that vitamin D deficiency or insufficiency should be corrected^13^.

Age is considered to be an important determinant of many clinical outcomes. According to the regression model, for each 1-year increase in age, the odds of achieving at least a 30% PTH reduction improved by 5.3%, with a corresponding 68% increase in the odds for each 1-decade increase in age. Of interest, it has been shown that cinacalcet reduced the risk of major cardiovascular events and death among HD patients older than 65 years^46^.

The high cost of cinacalcet may limit its use in clinical practice. Additional well-designed studies are needed to identify subgroups of patients for whom cinacalcet may potentially provide benefits by preventing SHPT or improving long-term clinical outcomes, and helping with the decision-making process in the management of patients with SHPT.

Our study has certain limitations. First, this is a retrospective observational study in a real clinical situation. Our ANCOVA model can explain approximately 35% of the variability in cinacalcet response, implying that there might be other factors, not examined in this study, that influence the variability of cinacalcet response in HD patients. Second, this study is a single-center study. However, the participants were uniformly treated with the same strategies for the management of SHPT. Third, this study focused on a 12-week treatment of cinacalcet; the possible association between the PTH lowering effect and clinical outcomes was not evaluated, given the fact that efficacy of cinacalcet in lowering PTH did not translate into a straightforward improved clinical outcome. According to the unadjusted intention-to-treat analysis of the Evaluation of Cinacalcet Hydrochloride Therapy to Lower Cardiovascular Events (EVOLVE) randomized controlled trial^47^, cinacalcet does not appear to provide a reduction in risk of mortality or major cardiovascular events in dialysis patients with moderate to severe SHPT. In clinical practice, cinacalcet is generally prescribed when a patient has a very high serum PTH concentration, and where considerable vascular damage may already have occurred. Given data from DOPPS suggesting that PTH levels > 600 pg/mL are associated with higher risk for all-causes and cardiovascular mortality and cardiovascular hospitalizations in dialysis patients^48^, the question of whether initiating cinacalcet treatment earlier in course of SHPT disease progression would prevent long-term unwanted clinical consequences remains to be answered. Finally, whether or not *CASR* genetic polymorphism would significantly impact the efficacy of a newer available calcimimetic agent remains an unanswered question.

In conclusion, *CASR* A990G polymorphism significantly influences the PTH lowering effect of cinacalcet treatment in HD patients with SHPT. The magnitude of PTH suppression and the proportion of patients achieving at least 30% PTH reduction from baseline to week 12 of cinacalcet treatment are greater in G carriers as compared to noncarriers.

## Methods

### Study design and participants

In this observational study, all adult patients (age > 18 years) who visited the King Chulalongkorn Memorial Hospital (KCMH) 1500-bed university hospital in Thailand, having been on HD for at least 3 months and having been treated with cinacalcet for SHPT for more than 12 weeks starting in the period of January 2015 to August 2019, were invited to participate. Patients with evidence of serious hepatic disease with a Child–Pugh score > 7, a history of previous surgical parathyroidectomy or percutaneous ethanol injection therapy into the parathyroid gland, incomplete record data, or those receiving strong inhibitors of cytochrome P450 enzyme that could interfere with cinacalcet pharmacokinetics were excluded. Cinacalcet was initiated at the dose of 25 mg per day at bedtime. Dietary restriction, the dose or type of phosphate binders or vitamin D sterols, and dialysate calcium concentration were adjusted at the discretion of the physicians according to standard of care.

The study protocol has been reviewed and approved by the Institutional Review Board of the Faculty of Medicine, Chulalongkorn University, Bangkok, Thailand. The IRB approval number is 672/61. The study was registered at Thai Clinical Trials Registry (TCTR20190820002) and was conducted in accordance with the declaration of Helsinki and its subsequent revisions. Each patient signed an informed consent document before enrollment.

### Data collection

Patient characteristics and relevant clinical information on the starting date of cinacalcet treatment, both as baseline data and at week 12 of cinacalcet treatment, were retrieved from KCMH electronic medical records. All serum biomarkers were measured at KCMH central laboratory. Serum iPTH levels were determined by using a chemiluminescence immunoassay on a Roche Elecsys 2010 Analyzer. Levels of 25-hydroxy vitamin D were assessed by using chemiluminescent immunoassay (CLIA, Diasorin, Italy). Throughout this study, serum calcium was reported as albumin corrected values when serum albumin was less than 4.0 g/dL [corrected calcium in mg/dL = measured calcium in mg/dL + 0.8*(4 – serum albumin in 4.0 g/dL)]^13^.

### Blood collection, DNA extraction and genotyping

Venous blood samples were collected into tubes containing EDTA during outpatient visits after recruitment. For genotyping, genomic DNA was extracted from the buffy coat that was collected from the peripheral blood using a QIAamp DNA Blood Mini kit for DNA purification (Qiagen, Hilden, Germany). The genotype analysis of *CASR* (c.2968A > G, rs1042636; assay ID: C_7504854_20) was performed using TaqMan® Assay Reagents for allelic discrimination (Applied Biosystems®, Foster City, CA, USA) with Applied Biosystem® QuantStudio 5 Real-Time PCR System (Thermo Fisher Scientific, Waltham, MA, USA). Each 20 μL PCR mixture contained 4 μL of genomic DNA (5 ng/μL), 10 μL of TaqMan® Genotyping Mastermix, 1 μL of allele specific TaqMan® MGB probe and sequence-specific primer kit, and 5 μL of DNase-free H_2_O. The conditions of PCR analysis were set as 50 °C for two minutes and 95 °C for 10 min, and then 40 cycles of 95 °C for 15 s and 60 °C for one minute.

Individual patients identified as either heterozygous or homozygous carriers of *CASR* 990G allele (the AG or GG genotypes) were classified as G carriers, and those identified as homozygous carriers of *CASR* 990A allele (the AA genotype) were classified as noncarriers. The physicians were not aware of the genotype of the patients at the time of any drug dosage modifications.

### Statistical analysis

Distribution of continuous data was assessed by Kolmogorov–Smirnov or Shapiro–Wilk test, and, subsequently, parametric tests or nonparametric tests were applied as appropriate. Continuous variables are presented as either means ± standard deviations when data are normally distributed, or medians with IQR when data are not normally distributed. Categorical data, on the other hand, are presented as counts and percentages. Continuous data were compared using the independent-samples t-test, analysis of variance (ANOVA), paired t-test, Wilcoxon Signed Rank test, Mann–Whitney U test, or Kruskal–Wallis test. Categorical variables were compared by chi-square test. Genotype frequencies of the polymorphism were tested for deviations from Hardy–Weinberg equilibrium using appropriate chi-square testing.

Assumptions for the ANCOVA and logistic regression were performed. Where appropriate, data were logarithmically transformed to obtain normal distribution. Bivariate correlations between continuous variables were evaluated by either Pearson correlation test or Spearman rank correlation test as appropriate. The linear relationships between continuous variables were assessed by simple linear regression analysis. The homogeneity of variances was tested by Levene’s test. The homogeneity of regression was analyzed by conducting a univariate ANOVA. The linear relationships between continuous independent variables and the logit transformation of the dependent variable were assessed by Box-Tidwell procedure.

The ANCOVA was used to evaluate the relationships between *CASR* genotypes and PTHw12. The potential covariates were selected based on the previous reports of clinical factors that significantly influenced PTH level^24,49,50^. The covariates considered were bPTH in *100 pg/mL, bPhos in mg/dL, bCa in mg/dL, bCtriol in μg/week, bErgo in *20,000 IU/month, and AGE in years. A relative calcitriol equivalent dose of alfacalcidol in μg/week was calculated by dividing the alfacalcidol dose per week by 1.5^51,52^. Any covariates that were strongly correlated with another covariate at *r* > 0.8 were eliminated, as they did not add a significant amount of information independent of the related variable. To indicate the degree to which each factor affected cinacalcet response, partial eta squared (ŋ_p_^2^), a measure of the effect size, was computed. Bootstrapping was performed to obtain 95% CI of the adjusted mean scores. The untransformed iPTH were also determined by backtransforming the adjusted means.

Binomial logistic regression was applied to compare the effect of *CASR* genotypes in terms of achievement of at least 30% reduction of iPTH levels from their baselines between the two studied groups whilst controlling for significant covariates. Overall model evaluation was assessed by omnibus tests of model coefficients. Significance of individual regression coefficients was tested by Wald chi-square statistic. The Hosmer–Lemeshow test and Nagelkerke R square were used to evaluate goodness-of-fit of a logistic model. Bootstrapping was also conducted to derive 95% CI of the odds ratio. The degree to which predictive probabilities, with a cutoff of 0.5, agree with actual outcomes was determined to document the validity of the predicted probabilities.

Power calculation was performed. With an effect size of 0.25, a power of 0.8, and a significant level of 0.05, a required total sample size of at least 128 persons was estimated by G*Power 3.1.9.2 software^53^. All statistical tests were performed using IBM SPSS version 22 (IBM, Bangkok Thailand). Unless stated otherwise, a two-sided p-value of less than 0.05 was considered statistically significant.

## Data Availability

The data that support the findings of this study are available from the corresponding author upon reasonable request.
